# Adapted Bacteriophages for Treating Urinary Tract Infections

**DOI:** 10.3389/fmicb.2018.01832

**Published:** 2018-08-07

**Authors:** Aleksandre Ujmajuridze, Nina Chanishvili, Marina Goderdzishvili, Lorenz Leitner, Ulrich Mehnert, Archil Chkhotua, Thomas M. Kessler, Wilbert Sybesma

**Affiliations:** ^1^The Alexander Tsulukidze National Center of Urology, Tbilisi, Georgia; ^2^The George Eliava Institute of Bacteriophage, Microbiology and Virology, Tbilisi, Georgia; ^3^Department of Neuro-Urology, Balgrist University Hospital, University of Zurich, Zurich, Switzerland

**Keywords:** bacteriophage therapy, Pyo bacteriophage, adaptation, urinary tract infection, antibiotic resistance

## Abstract

Urinary tract infections (UTIs) are among the most widespread microbial diseases and their economic impact on the society is substantial. The continuing increase of antibiotic resistance worldwide is worrying. As a consequence, well-tolerated, highly effective therapeutic alternatives are without delay needed. Although it has been demonstrated that bacteriophage therapy may be effective and safe for treating UTIs, the number of studied patients is low and there is a lack of randomized controlled trials (RCTs). The present study has been designed as a two-phase prospective investigation: (1) bacteriophage adaptation, (2) treatment with the commercially available but adapted Pyo bacteriophage. The aim was to evaluate feasibility, tolerability, safety, and clinical/microbiological outcomes in a case series as a pilot for a double-blind RCT. In the first phase, patients planned for transurethral resection of the prostate were screened (*n* = 130) for UTIs and enrolled (*n* = 118) in the study when the titer of predefined uropathogens (*Staphylococcus aureus*, *E. coli*, *Streptococcus* spp., *Pseudomonas aeruginosa*, *Proteus mirabilis*) in the urine culture was ≥10^4^ colony forming units/mL. *In vitro* analysis showed a sensitivity for uropathogenic bacteria to Pyo bacteriophage of 41% (48/118) and adaptation cycles of Pyo bacteriophage enhanced its sensitivity to 75% (88/118). In the second phase, nine patients were treated with adapted Pyo bacteriophage and bacteria titer decreased (between 1 and 5 log) in six of the nine patients (67%). No bacteriophage-associated adverse events have been detected. The findings of our prospective two-phase study suggest that adapted bacteriophage therapy might be effective and safe for treating UTIs. Thus, well-designed RCTs are highly warranted to further define the role of this potentially revolutionizing treatment option.

## Introduction

Emergence and re-emergence of multiple antibiotic resistant bacterial infections and their rapid spread in the environment has led to a new rise of scientific interest toward bacteriophage therapy as an alternative to antibiotics. Use of bacteriophages for treatment of bacterial infections has been suggested by the French-Canadian scientist Felix d’Herelle in 1917. Since then, bacteriophage therapy has been applied in different fields of medicine, for treatment of various bacterial infections (Chanishvili, 2012). However, after the discovery of penicillin in 1940s the Western scientific societies gave the preference to antibiotic therapy, while many physicians and researchers in the former Soviet Union republics remained dedicated to bacteriophage therapy and continued to use it alone or in combination with antibiotics (Chanishvili, 2012), see also **Supplementary Material** for more references, partly in Russian.

Lower urinary tract symptoms (LUTS) are a common problem in adult men with a high impact on quality of life (Martin et al., 2011). Traditionally LUTS have been related to bladder outlet obstruction, which is often caused by prostatic enlargement (Abrams et al., 2002). Prostatic enlargement occurs in about 25% of all men in their fifties, 30% in their sixties, and in 50% of men aged 80 years or older (Kupelian et al., 2006). Transurethral resection of prostate (TURP) is regarded the cornerstone of surgical treatment of LUTS secondary to benign prostatic obstruction (Cornu et al., 2015). These patients have a relevant risk for urinary tract infections (UTIs) (Schneidewind et al., 2017). Beside the possible development of residual urine, which acts as a growth medium for bacteria (Truzzi et al., 2008), many of these patients rely on a short or long-term catheterization prior to further treatment. Single insertion of a catheter causes infection in 1–2% of cases, while catheters with open-drainage systems result in bacteriuria in almost 100% of the cases within 3–4 days (Warren, 1992; Bonkat et al., 2018).

Therefore, we decided to combine TURP with bacteriophage therapy, using bacteriophages as a replacement of perioperative antibiotics. The present study has been designed as prospective two-phase (first phase: bacteriophage adaptation, second phase: treatment with the commercially available but adapted Pyo bacteriophage) study preceding a randomized, placebo-controlled, double-blind clinical trial (Leitner et al., 2017) to assess efficacy and safety of adapted bacteriophages for treating (catheter associated) UTIs (Nicolle et al., 2005; Hooton et al., 2010; Bonkat et al., 2018) in patients undergoing TURP.

## Patients and Methods

### Ethics Committee Approval

This prospective two-phase study has been approved by the local ethics committee (TNCU-02/283; Tbilisi, Georgia) and was conducted at the Alexander Tsulukidze National Center of Urology (TNCU), Tbilisi, Georgia and the Eliava Institute of Bacteriophage, Microbiology and Virology (EIBMV), Tbilisi, Georgia. The study was designed as an investigation preceding the randomized controlled trial (RCT) registered at ClinicalTrials.gov: NCT03140085 (Leitner et al., 2017).

### Patients

From September 2016, 130 patients planned for TURP were screened in preparation for the RCT (Leitner et al., 2017) at the TNCU. In the first phase, urine cultures from all patients (taken by mid-stream urine, or from the existing transurethral or suprapubic catheter) were evaluated. Overall, 118 (91%) of the 130 screened patients had positive urinary cultures with predefined uropathogens (i.e., *Staphylococcus aureus*, *E. coli*, *Streptococcus* spp., *Pseudomonas aeruginosa*, *Proteus mirabilis*) and ≥10^4^ colony forming units (CFU)/mL. The isolated cultures were consecutively subjected to an *in vitro* bacteriophage sensitivity test to the commercially available and in Georgia registered Pyo bacteriophage solution (Eliava BioPreparations Ltd., Tbilisi, Georgia), which underwent adaptation cycles, as described in the next paragraph. In the second phase, nine patients who had scored sensitive to the cocktail were further subjected to bacteriophage treatment in a non-blinded fashion. Exclusion criteria were symptomatic UTIs, microorganisms not sensitive to Pyo bacteriophage and age under 18 years. From all patients, prostate size, prostate specific antigen (PSA), International Prostate Symptom Score (IPSS) questionnaire (Barry et al., 1992) values, maximum flow rate and post void residual were collected prior to surgery. Resected prostate volume was collected and histological results were determined. Urine culture sampling was repeated 7 days after surgery or at the time of any adverse events. Written informed consent was obtained from all included patients.

### Bacteriophage Preparation and Adaptation

To cover a diversity of uropathogens a commercial preparation called Pyo bacteriophage produced by Eliava BioPreparations Ltd., Tbilisi, Georgia, was used for treating UTIs. This bacteriophage cocktail is composed of bacteriophage lines active against a broad spectrum of uropathogenic bacteria: *Staphylococcus aureus, E. coli, Streptococcus* spp. (including Streptococci group D renamed now to *Enterococcus* spp.), *Pseudomonas aeruginosa*, and *Proteus* spp. of urological infections (Chanishvili, 2012). As is common practice, commercial bacteriophage cocktails, including Pyo bacteriophage, are regularly adapted by the EIBMV with the aim to increase the efficacy of the bacteriophage cocktail toward newly emerging pathogens (Kutter et al., 2010; Villarroel et al., 2017; McCallin et al., 2018). Also in our study we applied adaptation to enhance the efficacy and coverage toward uropathogenic strains that initially scored intermediate or resistant in the *in vitro* sensitivity study, in a similar way as done in the previously conducted *in vitro* study (Sybesma et al., 2016). The method is based on Appelmans’ protocol for titration of bacteriophages (Appelmans, 1921) and selects for h-mutants with a broader and stronger host–bacteriophage interaction (Merabishvili et al., 2018). Similar as for the determination of the minimal inhibitory concentration for antibiotics (Levison and Levison, 2009), Appelmans’ method is based on liquid titration of bacteriophages and determines the lowest concentration of bacteriophages that show optical transparency over 24–72 h in a suspension with pre-selected bacterial strains resistant to the bacteriophage cocktail. This dilution with the lowest concentration of bacteriophages, cut-off point, is designated with negative degree values. If the initial bacteriophage titer was 10^-1^, it may become 10^-2^ or 10^-3^ with every dilution round, which indicates that more active bacteriophage units had been able to kill bacteria and that the tested bacteria had become less resistant to the adapted bacteriophages.

The subsequent titer of the bacteriophages is determined using two methods: titration in liquid (Appelmans, 1921) and titration using a double layer agar method (Gratia, 1936). The titration is done for each component included into Pyo bacteriophages separately on a standard set of host cultures (i.e., the titer of the *E. coli* bacteriophages is determined on the set of the standard *E. coli* strains, the titer of *Staphylococcus* component is determined on the set of the standard *Staphylococcus* strains, etc.). In this way, the titer of the bacteriophages in the range of 10^7^–10^9^ plaque forming units per mL (pfu/mL) is estimated. However, the titer of the individual (adapted) clones included into one group of bacteriophages may vary (McCallin et al., 2018).

### Microbiological Evaluation and Bacteriophage Sensitivity Test

**FIGURE 1:**
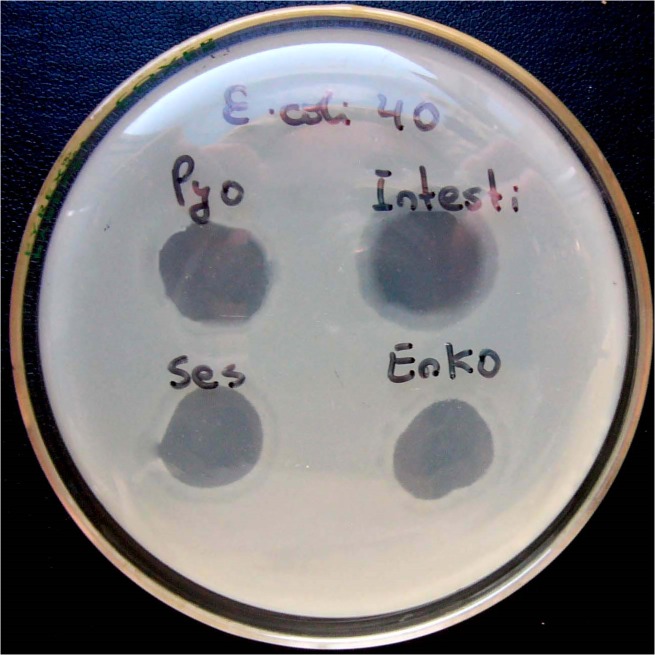
Different degrees of lyses of bacterial culture due to bacteriophage activity. The results in upper line (for Pyo bacteriophage and Intesti bacteriophage) are considered as “S” (sensitive), while the results in the lower line (Ses bacteriophage and Enko bacteriophage) are considered as “I” (intermediate). Note: Pyo, Intesti, Ses, and Enko bacteriophage are all commercially available bacteriophage cocktails. Picture was taken during previously conducted work (Sybesma et al., 2016) where several different bacteriophage cocktails were used.

In the first phase, urine samples were streaked in triple on the chromogenic Uriselect^TM^4 media (Bio-Rad Laboratories, Marnes-la-Coquette, France) for quantification and qualification of uropathogenic microorganisms. Positive urinary cultures were microscopically assessed regarding Gram stains and morphology. For all bacterial strains antibiotic and phage sensitivity tests were performed. If eligible microorganisms potentially treatable with Pyo bacteriophage (*S. aureus*, *E. coli, Streptococcus* spp., *P. aeruginosa, P. mirabilis*) were found, urine cultures were sent to the EIBMV and further screened for bacteriophage sensitivity. Hereto, the urine samples were re-cultivated and their identity was re-checked. As soon as the same eligible microorganisms had been cultivated a bacterial cell lysis screening assay was performed, as described previously (Sybesma et al., 2016). If *in vitro* results showed clear confluent lyses on the petri dish with the bacterial lawn, it was classified as sensitive (**Figure 1**). Resistant and intermediate resistant strains were used in adaptation cycles. In case of sensitivity Pyo bacteriophage was sent to the hospital to start the treatment. Seven days after TURP, urine samples were again collected and cultivated in triple on the above mentioned chromogenic Uriselect^TM^4 media and re-evaluated.

### Intravesical Bacteriophage Treatment

Transurethral resection of prostate was performed according general surgical practice using a monopolar resectoscope (May and Hartung, 2006). For low pressure irrigation, a suprapubic trocar was placed in every patient. No perioperative antibiotic prophylaxis was given. After TURP a suprapubic catheter and a transurethral catheter were placed to maintain irrigation. The transurethral catheter was removed after 24–48 h. The suprapubic catheter was kept in place for 7 days to enable adapted Pyo bacteriophage instillation. Pyo bacteriophage was instilled by a health care provider two times per 24 h (i.e., 8.00 h, 20.00 h) for 7 days, starting the first day after surgery. The solution of 20 mL was retained in the bladder for approximately 30–60 min.

### Assessment of Safety and Clinical/Microbiological Outcomes

All adverse events within the treatment phase were recorded as defined by the International Conference on Harmonisation (ICH) Good Clinical Practice (GCP) Guidelines (E6) (International Conference on Harmonisation, 1996) and International Organization for Standardization (ISO 14155) (International Organization for Standardization, 2011). Potential efficacy was assessed using clinical/microbiological parameters and defined as no clinical signs for infection and a reduction in CFU/mL.

### Outcome Parameters

Primary: (a) sensitivity of uropathogenic strains to the commercially available but adapted Pyo bacteriophage (first phase) and (b) effect of intravesical treatment with adapted Pyo bacteriophage (second phase).

Secondary: Occurrence/absence of adverse events, in categorization according to the National Cancer Institute Common Terminology Criteria for Adverse Events (CTCAE) version 4 in grade 1 to 5^1^ during bacteriophage treatment (second phase).

### Statistical Analyses

Descriptive statistics were used. Data are presented as percentages or mean ± standard deviation. Due to the limited number of subjects no further statistical analyses were performed.

## Results

### First Phase: *in vitro* Bacteriophage Sensitivity Testing and Adaptation Cycles

**FIGURE 2:**
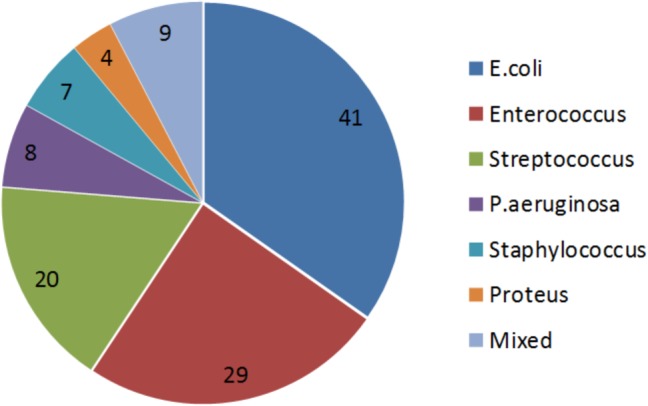
Proportional distribution of bacterial strains. Urinary cultures of the 118 included patients showed the following distribution of bacterial strains: *E. coli* was found predominantly with 41%, followed by *Enterococcus* spp. with 29%, *Streptococcus* spp. with 20%, *Pseudomonas aeruginosa* with 8%, *Staphylococcus* spp. 7%, *Proteus* spp. 4%, and others 9%.

**FIGURE 3:**
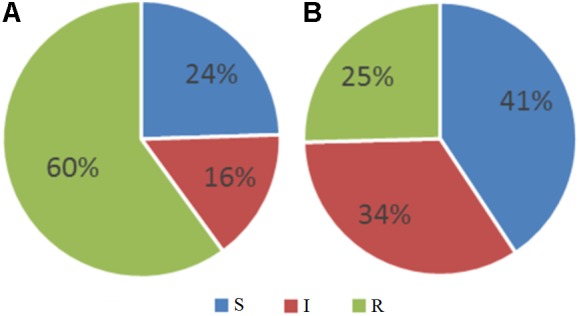
Proportional distribution of the sensitive, intermediate, and resistant strains among 118 clinical samples before **(A)** and after **(B)** four adaptation cycles of the Pyo bacteriophage cocktail.

The distribution of bacterial strains of the 118 included patients is shown in **Figure 2**. 24% and 17% of all strains were sensitive and intermediate sensitive to the initially used Pyo bacteriophage (i.e., total sensitivity of 41%), **Figure 3A**. After four adaptation cycles the sensitivity and intermediate sensitivity increased up to 41% and 34% (i.e., total sensitivity 75%), **Figure 3B**.

### Second Phase: Treatment With Adapted Pyo Bacteriophage

Patients characteristics are found in **Table 1**. The mean age was 69 ± 12 years, IPSS questionnaires revealed moderate to strong LUTS (IPSS 20 ± 2). The average prostate size was 77 ± 37 mL, all PSA values were within the non-pathological range. Maximum flow rate was 11 ± 3 mL/s with a mean post void residual of 80 ± 100 mL. Two patients relied on an indwelling catheter preoperatively. The average operation time was 48 min, no complications occurred during prostate surgery. Histological results revealed benign prostatic hyperplasia in all cases, five patients showed high grade prostatic intraepithelial neoplasia but no malignant disease was found.

**Table 1:** Summary of results of intravesical Pyo bacteriophage treatment conducted on nine patients.

Case	Age range (years)	Pre-treatment		Post-treatment		Prostate size (g)	PSA value (μg/L)	Adverse events/remarks
		Culture ID	Titer (CFU/mL)	Culture ID	Titer (CFU/mL)			
1	61–65	*E. coli*	10^7^	*E. coli*	10^5^	70	1	No adverse events
2	81–85	*E. coli*	10^7^	*E. coli*	10^4^	140	2	No adverse events
3	56–60	*E. coli*	10^4^	*E. coli*	10^4^	65	0.3	No adverse events
4	81–85	*E. coli*	10^7^	Non-pathogenic microflora	Not quantified	90	3.2	No adverse events
5	56–60	*Streptococcus*	10^7^	*Streptococcus*	10^6^	130	0.8	No adverse events
6	81–85	*Streptococcus*	10^5^	No bacterial growth	Below detection limit	29	2.3	No adverse events
7	66–70	*Enterococcus*	10^6^	*E. coli*	10^7^	45	1	Potential secondary infection
8	66–70	*Enterococcus*	10^6^	Non-pathogenic microflora	Not quantified	60	1.3	No adverse events
9	56–60	*Pseudomonas*	10^6^	No data	No data	60	1.3	Sudden fever and chills started on the 3rd day

Prior to treatment, urine culture revealed *E. coli* in four, *Streptococcus* spp. in two, *Enterococcus* spp. in two and *P. aeruginosa* in one of the nine patients. After treatment, four patients showed no significant bacterial growth, while *E. coli* and *Enterococcus* spp. were still isolated from the urine culture of four and one patient, respectively. In six out of nine patients (67%), bacterial titers decreased after bacteriophage treatment (**Table 1**).

No bacteriophage-associated adverse events have been detected. In one patient, an antibiotic therapy (third generation cephalosporin) was started at day 3 after development of fever (>38.0°C) and the symptoms disappeared within 48 h. Urine culture showed *P. aeruginosa.*

## Discussion

*In vitro* analysis showed a sensitivity for uropathogenic bacteria to the commercially available Pyo bacteriophage of 41%. Adaptation cycles of Pyo bacteriophage further enhanced its sensitivity to 75%. In our *in vivo* pilot series, the bacterial titers decreased after bacteriophage treatment in six out of nine patients (67%). No bacteriophage-associated adverse events have been detected but one patient developed fever due to *P. aeruginosa* infection with restitution of symptoms under antibiotic treatment.

Our study was designed as a feasibility, tolerability, and safety assessment and to evaluate clinical/microbiological outcomes of commercially available adapted Pyo bacteriophages preceding a placebo-controlled, double-blind RCT (Leitner et al., 2017). We have not investigated the composition of the continuously adapted Pyo bacteriophage cocktail by, e.g., metagenome analysis as recently described for previously used Pyo bacteriophage cocktails (Villarroel et al., 2017; McCallin et al., 2018), where it has also been reported that as a result of adaptation the titer of the individual bacteriophage clones included may vary. We expect that the detailed elucidation of the composition of bacteriophage cocktails as well as understanding the mechanisms behind the bacteriophage infection or bacterial resistance will become more relevant as soon as more conclusive outcomes about the efficacy of bacteriophage therapy has been described.

Bacteriophage therapy has already been practiced for decades in Eastern European countries (Chanishvili, 2012) and many people are aware of its existence (see also **Supplementary Material** for more references, partly in Russian). In the present open-label pioneering study for a placebo-controlled, double-blind RCT, the commercially available preparation Pyo bacteriophage was used for treating nine patients who were planned for TURP and had been diagnosed with UTI. Bacteriophage therapy only started after a positive result of an *in vitro* sensitivity analysis of the isolated uropathogen with the Pyo bacteriophage cocktail and did not cause any adverse events such as rise of body temperature, headache, hematuria, or allergic reaction in eight out of nine patients. Only in one case (# 9), on the 3rd day after prostate surgery, fever was observed. After a sudden onset of fever (38.5°C), the bacteriophage treatment was stopped, while a third generation cephalosporin was prescribed. In 48 h after the start of antibiotic therapy, the body temperature was normalized (24 h: 37.8°C; 48 h: <37.5°C). In this particular case the infection was caused by *P. aeruginosa*, which is known to release endotoxins during its lysis.

The secondary bacteriology testing of urine samples, taken after the bacteriophage treatment, demonstrated a positive tendency in therapy of infection, in particular a decrease of bacterial counts varying between 1 and 5 logs (cases # 1, 2, 5, 6). In one case (# 6) the secondary bacteriology analysis after bacteriophage therapy showed that urine had become below the detection limit of the Uriselect^TM^4 media (10^4^ CFU/mL for the uropathogens). In two cases (# 4, # 8) the initial infections, *E. coli* (titer 10^7^ CFU/mL) and *Enterococcus* (titer 10^6^ CFU/mL), respectively, had disappeared after bacteriophage therapy; however, presence of non-pathogenic micro-flora was observed which did not require any further treatment. It is notable that in these two cases the non-pathogenic flora appeared in aged patients 69–80 years old, which may be a result of urination difficulties remaining even after the operation. In one case (# 3) the titer of *E. coli* did not change after the bacteriophage treatment. In case (# 7) the initial infection caused by *Enterococcus* (titer 10^6^ CFU/mL) after bacteriophage therapy was replaced by *E. coli* (titer 10^7^ CFU/mL), which may be attributed to a secondary infection.

Although the design and number of cases and the diversity of the results described in this publication do not permit to draw out any statistically reliable conclusions, the trend indicated by the data from our study does not stand on its own and corresponds well with the outcome of several other recently reported cases where bacteriophage therapy was used in Western countries (Abedon et al., 2017). In terms of safety, the findings of our prospective two-phase study support earlier made conclusions that bacteriophage therapy using broad spectrum bacteriophages cocktails, including Pyo bacteriophage, is safe (McCallin et al., 2013, 2018; Sarker et al., 2016). However, for a definite conclusion about efficacy of bacteriophage treatment, well designed RCTs are urgently needed.

Due to a too high use of antibiotics in today’s society, the emergence of antibiotic resistance pathogens has become a serious problem in terms of increased morbidity and mortality rates as well as the elevated healthcare costs as has been brought to the public attention by several national and international health protection agencies (CDC, 2013; European Centre for Disease et al., 2017; Leitner et al., 2017). Since the resistance mechanism of bacteria against bacteriophages differs from those against antibiotics, and since bacteriophage are self-replicating and self-evolving entities, bacteriophage therapy could be used as an alternative method to eliminate antibiotic resistant bacteria. One of the main limitations for acceptance and reimplementation of bacteriophage therapy is the lack of placebo-controlled, double-blind RCTs in agreement with Western standards (Expert round table on acceptance and re-implementation of bacteriophage therapy, 2016). We expect that the RCT we preceded with the present open-label study will contribute to conclude on the efficacy, cost and benefits of bacteriophages in case of antibiotic resistant uropathogenic bacteria.

Finally, we would like to remark that before bacteriophages can become accepted and broadly applied for treatment of certain bacterial infections, as is already practiced in several Eastern European countries, the legislative framework in the Western world needs to be adjusted. Since the intrinsic strength of bacteriophages relates to their antagonistic evolution potential with their bacterial hosts, the composition of effective bacteriophages cocktails will not be static, but adapted and adjusted over time, which assures efficacy toward evolving bacterial infections at different moments at different places for different groups of patients. However, such a dynamic approach is not compatible with today’s production and admission requirements for chemical drugs. Although the use of bacteriophages is already quite old, it is remarkable to acknowledge that in fact a more tailor-made development and application of bacteriophages are in line with the increasing needs and opportunities around personalized nutrition and personalized medicine. A recent breakthrough in this debate has been reported for Belgium, where the national authorities agreed on setting up a practical bacteriophage therapy framework that relates on the magistral preparation (compounding pharmacy in the United States) of custom-made bacteriophage medicines (Pirnay et al., 2018). This Belgian “magistral bacteriophage medicine” framework is expected to be flexible enough to exploit and further explore the specific nature of bacteriophages as co-evolving antibacterials whilst giving precedence to patients’ safety.

## Conclusion

In our prospective two-phase study preceding a placebo-controlled, double-blind RCT, adaptation cycles enhanced the *in vitro* sensitivity of 118 strains to the commercially available Pyo bacteriophage from 41% to 75%. In the *in vivo* pilot series, a promising clinical and microbiological effect and excellent tolerability of adapted Pyo bacteriophage treatment could be shown. Our findings suggest that bacteriophage therapy might be effective and safe for treating UTIs. Thus, well-designed RCTs are highly warranted to further define the role of this potentially revolutionizing treatment option.

## Author Contributions

All authors contributed in designing and setting up the clinical study. AU conducted the bacteriophage treatment. NC and MG conducted all work related to bacteriophages. NC and WS drafted the manuscript. AU, MG, UM, and AC critically reviewed the manuscript. LL and TK made the final editing to the manuscript. All the authors read and approved the final manuscript.

## Conflict of Interest Statement

The authors declare that the research was conducted in the absence of any commercial or financial relationships that could be construed as a potential conflict of interest.

## Footnotes

1 http://ctep.cancer.gov/protocolDevelopment/electronic_applications/ctc.htm
