# Supplementary material for: Adapted Bacteriophages for Treating Urinary Tract Infections
Source: Front Microbiol. 2018 Aug 7;9:1832. doi: 10.3389/fmicb.2018.01832 (PMC6090023; doi:10.3389/fmicb.2018.01832)
Supplement: Supplementary file 1 [file Data_Sheet_1.DOCX]

**SUPPLEMENTARY MATERIAL**

**LIST OF ADDITIONAL REFERENCES (partly in Russian)**

References 1-11 report on application of bacteriophage therapy in different fields of medicine, including surgery, pediatrics, oto-laryngology, ophthalmology, gynecology, pediatrics, dermatology, surgery, traumatology, epidemiology, gastrointestinal diseases, for treatment of various bacterial infections, such as *Staphylococcus, Streptococcus, Pseudomonas, Proteus, E. coli, Shigella, Salmonella*, and many more.

References 12-25 specifically report on the application of bacteriophage therapy in urology.

1. Slavinsky A.N., Pshonkina D.M.,Svistushkin V.M., Bacteriophages in the complex treatment of acute bacterial rinosynusitis, 2014, Russian Medical Journal, 26:19. <http://www.rmj.ru/articles/otorinolaringologiya/Bakteriofagi_v_kompleksnom_lechenii_ostrogo_bakterialynogo_rinosinusita/#ixzz4xiwxW7Y0>
2. Kilasonia T, Karanadze N., 2001, Treatment of acute conjunctivitis with Pio-bacteriophage among newborns and infants, Selected Articles of the Tbilisi medical University, v. 36, p.248-250.,
3. Arshba, C., and Bagdoeva, K. (1965). Application of phage therapy against colibacterial infections of urinary tract. *Clinical and Experimental Urology* 3**,** 7-13.
4. Bochorishvili E. V. Chanishvili T.G., Sharashidze T.G., Nadiradze M.M., 1988, Method of treatment of the tube infertility of the inflammatory nature, Patent SU 1395331 A 1, Authors Certificate # 1395331;
5. Samsygina G.A., Boni E.G., 1984, Bacteriophages and phage therapy in pediatric practice, Pediatrics, # 4, pp. 67-68.
6. Vartapetov A.Y., 1957, Bacteriophage therapy of deep forms of staphylococcal dermatitis, In Book: “Bacteriophage Re­search”, Selected articles of Inter-Institutional Conference taking place in Tbilisi on October, 26-29, 1955, pp.411-426.
7. Chkhetia N., 1984, Treatment of lung diseases.Cand. Diss, Tbilisi, Georgia.
8. Meladze D.G., Mebuke M.G., Chkhetia N.Sh., Kiknadze N.Y., Koguashvili G.G., Timoshuk I.I., Larionova N.G., Vasadze G.K., 1981, Effect of the Staphylococcal bacteriophgage for treatment of purulent infections of lungs and pleura., Breast Surgery (“Grudnaya Khirurgia”), # 1, pp.53-56.
9. Tavebridze L.N., 1993, Post-traumatic treatment of the long bones, Cand. Diss., Tbilisi, Georgia.
10. Kurochka V.K., Karniz A.F., Khodyrev A.P., 1987, Experiences of implementation of preventive anti-epidemic measures in the center of intestinal infections with water transmission mechanism of morbidity, Military-Medical Journal, N 7, pp. 36-37.
11. Nesterova G.N. & Alexeeva N.A., 1961, To the issue of application of Coli-Proteus bacteriophage for treatment of colienteritis among young children, Selected Articles, Gorki Research Institute of Epidemiology and Microbiology, Issue VII, Intesinal diseases, pp. 312- 317.
12. Tsulukidze A.P., 1938, Application of phages in urology, Urology (“Urologia”), v. XV, N 1, pp.10-13.
13. Kolomintsev, N.B., Goroeinko, I.I., Shakmatov, V.K., and Brazhnik, P.K., 1966, Treatment of urological infections with different antibiotics and specific coli-proteus bacteriophages. In: Strategy and tactics of antibiotic therapy. Krasnodar (Ed.), 172-174.
14. Danilova, T.M., 1996, Phage therapy of the inflammatory urogenital infections in women. Proceedings of Dermatology and Venerology 5, 75.
15. Perepanova T.S. 1996, Comprehensive treatment and prevention of hospital infection of urine ways, Cand. Diss
16. Perepanova T.S., Debreeva O.S., Koliatarova G.A., Kondratieva E.M., Aiskaia I.M., Malysheva V.F., Baiguzina F.A., Grishkova N.Y., 1997, The efficacy of bacteriophage preparations in treating inflammatory urogenital diseases, Urology & Nephrology, Sept-Oct., # 5, 14- 7.
17. Parfenchuk R.L., 2004, Microbiological basis of oral phagotherapy of purulent-inflammatory diseases, Cand. Diss
18. Prokopenko E.I., 2005, Diagnosis, complex treatment and prevention of infectious complications in recipients of the kidney transplant, Cand. Diss
19. Lasareva E.B.m 2007, Bacteriophages and pectins in correction of microbiocenosis disorders in purulent-inflammatory processes, Cand. Diss
20. Belopolsky A.A. 2008, Ways to optimize the treatment of patients with acute cystitis in ambulatory practice, Cand. Diss.
21. Makarova E.K., 2008, Clinical and biochemical studies in pregnant women with pyelonephritis in the treatment of bacteriophage, Cand. Diss.
22. Musin D.R., 2008, Prophylaxis and treatment of complications of urolithiasis after contact lithotripsy in infected urolithiasis, Cand. Diss.
23. Letkiewicz, S., Miedzybrodzki, R., Klak, M., Jonczyk, E., Weber-Dabrowska, B., and Gorski, A. (2010). The perspectives of the application of phage therapy in chronic bacterial prostatitis. FEMS Immunol Med Microbiol 60, 99-112.
24. Kutter, E., De Vos, D., Gvasalia, G., Alavidze, Z., Gogokhia, L., Kuhl, S., and Abedon, S.T. (2010). Phage therapy in clinical practice: treatment of human infections. Curr Pharm Biotechnol 11, 69-86.
25. Khawaldeh, A., Morales, S., Dillon, B., Alavidze, Z., Ginn, A.N., Thomas, L., Chapman, S.J., Dublanchet, A., Smithyman, A., and Iredell, J.R. (2011). Bacteriophage therapy for refractory Pseudomonas aeruginosa urinary tract infection. J Med Microbiol 60, 1697-1700.
